# Sugammadex-Enhanced Neuronal Apoptosis following Neonatal Sevoflurane Exposure in Mice

**DOI:** 10.1155/2016/9682703

**Published:** 2016-11-08

**Authors:** Maiko Satomoto, Zhongliang Sun, Yushi U. Adachi, Koshi Makita

**Affiliations:** ^1^Department of Anesthesiology, Graduate School of Medical and Dental Sciences, Tokyo Medical and Dental University, Tokyo 1138519, Japan; ^2^Department of Anesthesiology, Graduate School of Medicine, Nagoya University, Aichi, Japan

## Abstract

In rodents, neonatal sevoflurane exposure induces neonatal apoptosis in the brain and results in learning deficits. Sugammadex is a new selective neuromuscular blockade (NMB) binding agent that anesthesiologists can use to achieve immediate reversal of an NMB with few side effects. Given its molecular weight of 2178, sugammadex is thought to be unable to pass through the blood brain barrier (BBB). Volatile anesthetics can influence BBB opening and integrity. Therefore, we investigated whether the intraperitoneal administration of sugammadex could exacerbate neuronal damage following neonatal 2% sevoflurane exposure via changes in BBB integrity. Cleaved caspase-3 immunoblotting was used to detect apoptosis, and the ultrastructure of the BBB was examined by transmission electron microscopy. Exposure to 2% sevoflurane for 6 h resulted in BBB ultrastructural abnormalities in the hippocampus of neonatal mice. Sugammadex alone without sevoflurane did not induce apoptosis. The coadministration of sugammadex with sevoflurane to neonatal mice caused a significant increase (150%) in neuroapoptosis in the brain compared with 2% sevoflurane. In neonatal anesthesia, sugammadex could influence neurotoxicity together with sevoflurane. Exposure to 2% sevoflurane for 6 h resulted in BBB ultrastructural abnormalities in the hippocampus of neonatal mice.

## 1. Introduction

Sugammadex is a new selective neuromuscular blockade (NMB) binding agent [1, 2]. It is used only to reverse aminosteroid muscle relaxants [2]. It is composed of eight sugar molecules in a doughnut shape, and it has been formulated to encapsulate NMB into its cavity in an irreversible form [3]. Sugammadex is used by anesthesiologists to achieve immediate reversal of an NMB with few side adverse effects. Recently, sugammadex was shown to induce neuronal apoptosis in primary cultures [4].

Due to its charge and high molecular weight (2178), it is thought that sugammadex cannot pass through the blood brain barrier (BBB) and placenta [5]. However, it may cross the BBB more easily under different clinical conditions such as brain injury [6] and sepsis [7, 8] and in the immature central nervous system [9].

Volatile anesthetics can influence BBB opening and integrity in aged rats [10, 11] and in brain injury [12]. Volatile anesthetics at clinical concentrations induced neuronal apoptosis and cognitive disorders in neonatal and aged rodents [13–19]. We previously studied neuronal apoptosis and cognitive dysfunction in mice with neonatal sevoflurane exposure [14, 19]. That neuronal damage was due to exposure of the immature central nervous system [20].

The developing brain is vulnerable to sevoflurane exposure [14, 15, 19]. We postulated that sugammadex coadministration with neonatal sevoflurane may exacerbate neuronal apoptosis due to changes in BBB integrity. Therefore, in this study, we investigated whether neonatal sevoflurane exposure altered the integrity of the BBB and whether the intraperitoneal administration of sugammadex enhanced the neuronal damage associated with neonatal 2% sevoflurane exposure.

## 2. Materials and Methods

The Animal Care and Use Committee of Tokyo Medical and Dental University approved the current study protocol (0160072A).

### 2.1. Animals

Postnatal day-5 C57BL/6 mice (males and females, mean body weight: 2.7 g) were purchased as a litter with their mother (SLC Japan, Shizuoka, Japan). The mice were housed under a 12 h light : dark cycle (lights on from 08:00 to 20:00), and room temperature was maintained at 21 ± 1°C. The same number of pups from each litter was used for the experiments to reduce variability related to the use of different litters (total number of pups used is 48, same number of males and females). All mice had* ad libitum* access to water and food.

Pups from the same litter were divided randomly into four groups: (1) nonanesthesia (NA group, control mice), (2) sugammadex treatment (sugammadex group, in which mice received an intraperitoneal injection of sugammadex), (3) 2% sevoflurane exposure (SEVO group, in which mice received 2% sevoflurane for 6 h), and (4) sugammadex combined with sevoflurane (sugammadex + SEVO group, in which mice received 2% sevoflurane for 6 h and an intraperitoneal injection of sugammadex).

### 2.2. Anesthesia Treatment

Sevoflurane anesthesia was performed as described previously [19]. Briefly, postnatal day-6 mice were placed in a humid chamber immediately after removal from the maternal cage. Sevoflurane (2%) was administered for 6 h with 40% oxygen as the carrying gas. Control mice were exposed to 40% oxygen. Total gas flow was 1 L/min.

### 2.3. Sugammadex Treatment

Sugammadex was dissolved in phosphate-buffered saline (PBS) and injected intraperitoneally with a single 30 mg/kg dose. Injection volume was 10 *μ*L. The same volume of PBS was administered to control animals. Sugammadex was administered intraperitoneally 30 min before induction with 2% sevoflurane anesthesia.

### 2.4. Western Blot Analysis

Western blot analysis of the forebrain protein lysate was performed as described previously [19]. The forebrains were removed quickly after 6 h of sevoflurane anesthesia. Primary antibodies included anti-cleaved caspase-3 (#9661, rabbit polyclonal, Cell Signaling Technology, Beverly, MA, USA), anti-cleaved poly[adenosine diphosphate-ribose] polymerase (PARP) (#9544, rabbit polyclonal, Cell Signaling Technology), and anti-*β*-actin (AC-15, mouse monoclonal, Sigma, St. Louis, MO, USA). Secondary antibodies included horseradish peroxidase (HRP)-linked anti-rabbit IgG (ab98493, donkey polyclonal, Abcam, Cambridge, MA, USA) and HRP-linked anti-mouse IgG (NA9310, sheep, GE Healthcare, Little Chalfont, England). A mouse *β*-actin antibody was used as the loading control. The protein bands were visualized using a chemiluminescence detection system (SuperSignal West Pico; Pierce, Rockford, IL, USA).

### 2.5. Immunohistochemistry

Immunohistochemistry was performed as described previously [19]. The tissues were perfused after 6 h of sevoflurane anesthesia. Briefly, vibratome sections (50 *μ*m thick) were washed with PBS containing 0.3% Triton X-100, and endogenous peroxidase activity was blocked with 1% H_2_O_2_ applied for 30 min. Next, the sections were incubated overnight at 4°C with anti-cleaved caspase-3 (#9661, rabbit polyclonal, Cell Signaling Technology) diluted in 1% blocking reagent (Roche, Basel, Switzerland) in TBS/Tween 20. Secondary antibodies included HRP-linked anti-rabbit IgG (ab98493, donkey polyclonal, Abcam).

Cleaved caspase-3 signal was visualized with a DAB-nickel substrate (0.05% DAB, 0.05% NiSO_4_, 0.015% H_2_O_2_, 0.05% 1 M Tris-HCl, pH 6.7). The sections were mounted on slides and counterstained with hematoxylin.

### 2.6. Evaluation of BBB Integrity

The tissues were perfused with freshly prepared paraformaldehyde (4%) and glutaraldehyde (2.5%) after 6 h of sevoflurane anesthesia. The brains were removed and the hippocampus CA1 region was cut and stored in the same fixative overnight at 4°C. After dehydration through a graded methanol series, the brain regions were embedded in Epon 812 and polymerized overnight at 60°C. Ultrathin (90 nm) sections were cut with a microtome, double stained with uranyl acetate and lead citrate according to standard procedures [21], and examined by transmission electron microscopy (H-7100; Hitachi, Ibaragi, Japan).

### 2.7. Statistical Analysis

Data are expressed as mean ± SEM. The protein expression levels detected by Western blot analysis are expressed as a percentage of the control (NA group). The statistical analysis was performed using SPSS statistical software package (SPSS ver. 24.0, IBM, Armonk, NY, USA). One-way analysis of variance (ANOVA) followed by the* post hoc* Newman-Keuls multiple comparison test was used for comparisons. *P* value < 0.05 was considered statistically significant.

## 3. Results

### 3.1. Ultrastructure of the BBB

**Figure 1:**
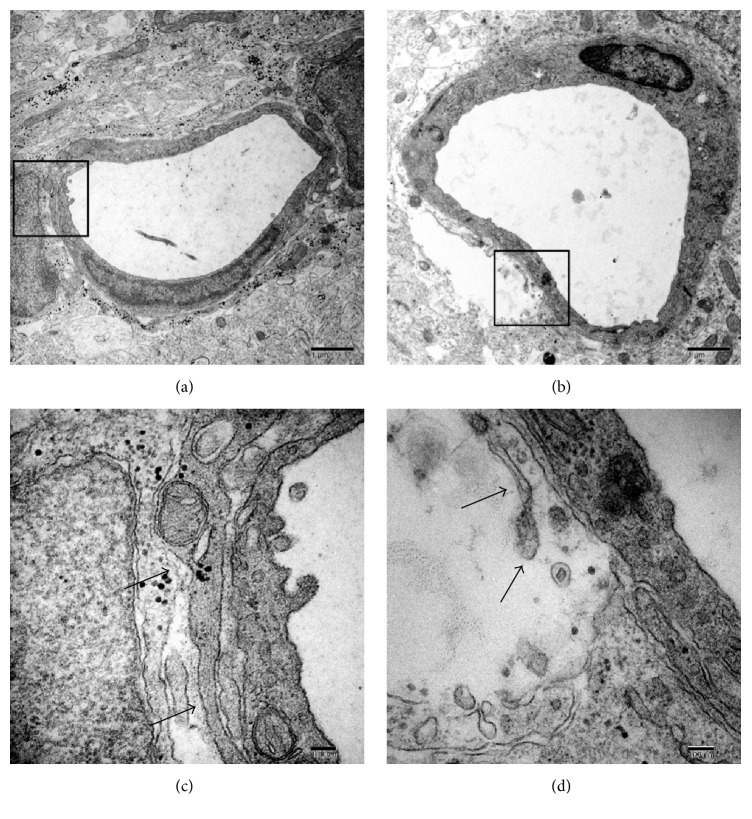
Ultrastructure of the capillaries in the hippocampus CA1 region in neonatal mice of the (a, c) control and (b, d) sevoflurane exposure groups. In the control group (a, c), the capillaries were continuous and integrated (black arrows), and the perivascular spaces were normal. In comparison, the ultrastructural integrity of the BBB appeared to be disrupted (black arrows) as a result of enlarged perivascular spaces after 6 h of sevoflurane exposure (b, d). Higher magnification views of the contacts (black boxes in (a) and (b)) are presented in (c) and (d). Scale bars: 1 *μ*m (a, b); 100 nm (c, d).

In the control group, the ultrastructure of the capillaries was continuous and integrated (Figure 1(a)), and the endothelial and perivascular spaces were normal. In comparison, the ultrastructural integrity of the BBB appeared to be disrupted, and the perivascular spaces were enlarged after 6 h of sevoflurane exposure (Figure 1(b)). In summary, 2% sevoflurane exposure for 6 h led to disruption of BBB ultrastructure in P6 mice.

### 3.2. Apoptosis

**Figure 2:**
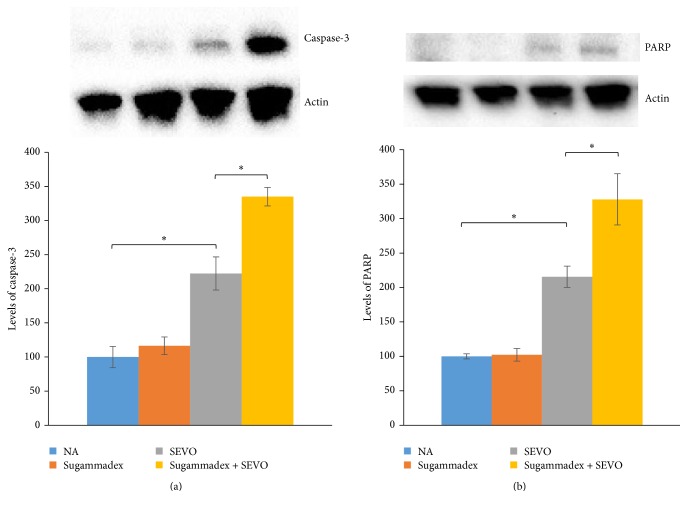
Apoptosis in the forebrain was evaluated by (a) cleaved caspase-3 and (b) cleaved PARP activation in each group after 6 h of sevoflurane exposure. Exposure to sevoflurane for 6 h increased neuronal apoptosis compared to the control group. Coadministration of sugammadex significantly exacerbated neuronal apoptosis induced by sevoflurane exposure. Sugammadex alone did not cause apoptosis (^*∗*^ *P* < 0.05, *n* = 8 each).

**Figure 3:**
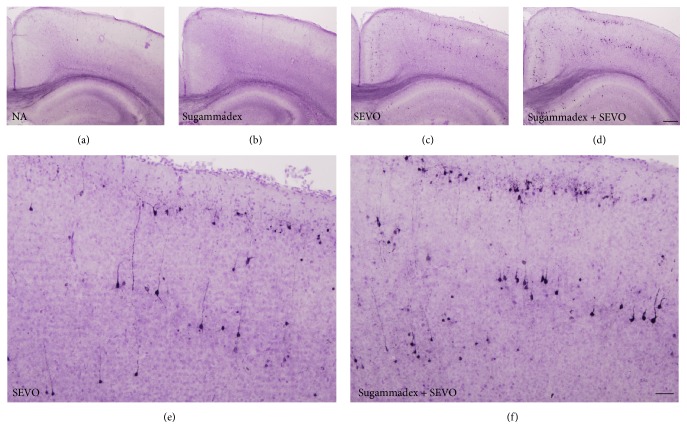
Cleaved caspase-3 activation in the retrosplenial cortex of the brain. Black dots indicate cleaved caspase-3-positive cells in the (a) nonanesthesia (NA), (b) sugammadex, (c, e) sevoflurane (SEVO), and (d, f) sugammadex + SEVO groups. Scale bars: 200 *μ*m (a–d); 50 *μ*m (e, f). Six hours of neonatal sevoflurane exposure increased the number of cleaved caspase-3-positive cells, indicating apoptosis. The intraperitoneal administration of sugammadex significantly enhanced neuronal apoptosis associated with neonatal 2% sevoflurane exposure.

Neonatal sevoflurane exposure for 6 h increased the concentration of cleaved caspase-3 (Figures 2(a) and 3) and another apoptosis marker, cleaved PARP (Figure 2(b)), compared with controls in P6 mice immediately after exposure. Sugammadex alone did not cause neuronal apoptosis. Interestingly, the intraperitoneal administration of sugammadex significantly enhanced neuronal apoptosis associated with neonatal 2% sevoflurane exposure (Figures 2 and 3). The increase in apoptosis was robust, and the apoptotic response to combined sugammadex and 2% sevoflurane exposure followed the pattern reported for other anesthetic drugs and ethanol [13–15, 22].

## 4. Discussion

Exposure to 2% sevoflurane for 6 h resulted in BBB ultrastructural abnormalities in the hippocampus of neonatal mice. Furthermore, this study shows that the coadministration of sugammadex and sevoflurane to neonatal mice caused a significant increase in neuroapoptosis in the brain compared with exposure to 2% sevoflurane alone.

Sugammadex itself has no anesthetic effect and by itself did not cause neuronal apoptosis without neonatal sevoflurane exposure in this in vivo model. Volatile anesthetics can influence BBB opening and integrity in aged rats [10, 11]. In this study, we confirmed disruption of the BBB ultrastructure in neonatal mice exposed to sevoflurane. This study indicated that sugammadex passed through the BBB, which was disrupted by neonatal sevoflurane exposure, and that sugammadex induced neuronal apoptosis as seen in primary culture [4].

The sugammadex concentration achieved by intraperitoneal injection was slightly higher than that in clinical use in human patients (from 2 to 16 mg/kg, intravenous injection for humans). In this study, we chose 30 mg/kg because undiluted sugammadex could be used in most pups. Sugammadex is not metabolized and is eliminated only via the urine. Sevoflurane anesthesia is one of the most commonly used volatile anesthetics for the induction and maintenance of general anesthesia during surgery. During surgery, including pediatric surgery, sugammadex is administered the anesthesiologist to reverse the NMB. Therefore, sugammadex and sevoflurane are present together during surgery. Nevertheless, because sugammadex forms a 1 : 1 complex with steroidal NMB drugs, most of the sugammadex remains free in the extracellular fluid. Sugammadex and sevoflurane might induce adverse effect on the brain.

We previously investigated neonatal sevoflurane neurotoxicity [14, 19] and found that 3% sevoflurane exposure for 6 h did not induce hypoxia or hypoventilation [14, 15]. Inhalation anesthetics show dose-dependent respiratory and circulatory depressive effects. Therefore, 2% sevoflurane is a safe concentration for both human children and mouse pups.

However, further study is required to investigate the mechanism by which sugammadex causes neuronal apoptosis. Palanca et al. [4] reported that sugammadex altered cholesterol homeostasis. Long-term memory impairment must be examined in a future study because we previously showed that high levels of apoptosis soon after anesthesia cause long-term memory impairment in adults [14, 19]. In addition, it will be necessary to investigate which concentrations of sugammadex and period of sevoflurane inhalation have the most harmful effects on the developing brain. Furthermore, it will be necessary to investigate measures to prevent neuronal apoptosis.

## 5. Conclusions

The results of this study suggest that sugammadex coadministered with neonatal sevoflurane exposure enhances neuronal apoptosis. Exposure to 2% sevoflurane for 6 h resulted in BBB ultrastructural abnormalities in the hippocampus of neonatal mice.

## References

[1] Nicholson W. T., Sprung J., Jankowski C. J. (2007). Sugammadex: a novel agent for the reversal of neuromuscular blockade. *Pharmacotherapy*.

[2] Naguib M. (2007). Sugammadex: another milestone in clinical neuromuscular pharmacology. *Anesthesia and Analgesia*.

[3] Chambers D., Paulden M., Paton F. (2010). Sugammadex for reversal of neuromuscular block after rapid sequence intubation: a systematic review and economic assessment. *British Journal of Anaesthesia*.

[4] Palanca J. M., Aguirre-Rueda D., Granell M. V. (2013). Sugammadex, a neuromuscular blockade reversal agent, causes neuronal apoptosis in primary cultures. *International Journal of Medical Sciences*.

[5] Farrar J. T. (2008). *Meeting of the Anesthetic and Life Support Drugs FDA Advisory Committee*.

[6] Obermeier B., Daneman R., Ransohoff R. M. (2013). Development, maintenance and disruption of the blood-brain barrier. *Nature Medicine*.

[7] Deng Y. Y., Fang M., Zhu G. F., Zhou Y., Zeng H. K. (2013). Role of microglia in the pathogenesis of sepsis-associated encephalopathy. *CNS and Neurological Disorders—Drug Targets*.

[8] Esen F., Erdem T., Aktan D. (2005). Effect of magnesium sulfate administration on blood-brain barrier in a rat model of intraperitoneal sepsis: a randomized controlled experimental study. *Critical Care*.

[9] Saunders N. R., Knott G. W., Dziegielewska K. M. (2000). Barriers in the immature brain. *Cellular and Molecular Neurobiology*.

[10] Hu N., Guo D., Wang H. (2014). Involvement of the blood-brain barrier opening in cognitive decline in aged rats following orthopedic surgery and high concentration of sevoflurane inhalation. *Brain Research*.

[11] Cao Y., Ni C., Li Z. (2015). Isoflurane anesthesia results in reversible ultrastructure and occludin tight junction protein expression changes in hippocampal blood-brain barrier in aged rats. *Neuroscience Letters*.

[12] Thal S. C., Luh C., Schaible E.-V. (2012). Volatile anesthetics influence blood-brain barrier integrity by modulation of tight junction protein expression in traumatic brain injury. *PLoS ONE*.

[13] Jevtovic-Todorovic V., Hartman R. E., Izumi Y. (2003). Early exposure to common anesthetic agents causes widespread neurodegeneration in the developing rat brain and persistent learning deficits. *The Journal of Neuroscience*.

[14] Satomoto M., Satoh Y., Terui K. (2009). Neonatal exposure to sevoflurane induces abnormal social behaviors and deficits in fear conditioning in Mice. *Anesthesiology*.

[15] Kodama M., Satoh Y., Otsubo Y. (2011). Neonatal desflurane exposure induces more robust neuroapoptosis than do isoflurane and sevoflurane and impairs working memory. *Anesthesiology*.

[16] Xiong W.-X., Zhou G.-X., Wang B. (2013). Impaired spatial learning and memory after sevoflurane-nitrous oxide anesthesia in aged rats is associated with down-regulated cAMP/CREB signaling. *PLoS ONE*.

[17] Li X.-M., Su F., Ji M.-H. (2014). Disruption of hippocampal neuregulin 1-ErbB4 signaling contributes to the hippocampus-dependent cognitive impairment induced by isoflurane in aged mice. *Anesthesiology*.

[18] Luo X., Yang L., Chen X., Li S. (2014). Tau hyperphosphorylation: a downstream effector of isoflurane-induced neuroinflammation in aged rodents. *Medical Hypotheses*.

[19] Sun Z., Satomoto M., Adachi Y. U., Kinoshita H., Makita K., Galley H. F. (2016). Inhibiting NADPH oxidase protects against long-term memory impairment induced by neonatal sevoflurane exposure in mice. *British Journal of Anaesthesia*.

[20] Edwards D. A., Shah H. P., Cao W., Gravenstein N., Seubert C. N., Martynyuk A. E. (2010). Bumetanide alleviates epileptogenic and neurotoxic effects of sevoflurane in neonatal rat brain. *Anesthesiology*.

[21] Ichinose S., Muneta T., Koga H. (2010). Morphological differences during in vitro chondrogenesis of bone marrow-, synovium-MSCs, and chondrocytes. *Laboratory Investigation*.

[22] Wozniak D. F., Hartman R. E., Boyle M. P. (2004). Apoptotic neurodegeneration induced by ethanol in neonatal mice is associated with profound learning/memory deficits in juveniles followed by progressive functional recovery in adults. *Neurobiology of Disease*.

