# Corrigendum to “Sugammadex-Enhanced Neuronal Apoptosis following Neonatal Sevoflurane Exposure in Mice”

**DOI:** 10.1155/2017/1368514

**Published:** 2017-10-17

**Authors:** Maiko Satomoto, Zhongliang Sun, Yushi U. Adachi, Koshi Makita

**Affiliations:** ^1^Department of Anesthesiology, Graduate School of Medical and Dental Sciences, Tokyo Medical and Dental University, Tokyo 1138519, Japan; ^2^Department of Anesthesiology, Graduate School of Medicine, Nagoya University, Aichi, Japan

In the article titled “Sugammadex-Enhanced Neuronal Apoptosis following Neonatal Sevoflurane Exposure in Mice” [1], the concentration of sugammadex injected into the mice, 30 mg/kg, was incorrect; the correct concentration is 300 mg/kg. The undiluted sugammadex concentration was 100 mg/ml and not 10 mg/ml. Accordingly, in the section “Sugammadex Treatment,” the statement “with a single 30 mg/kg dose” should be corrected to “with a single 300 mg/kg dose” and, in the Discussion section, the statement “we chose 30 mg/kg” should be corrected to “In this study, we chose 300 mg/kg.”
